# Empowering Medical Students to Respond to Discriminatory Comments from Patients: A Just-in-Time Training Method

**DOI:** 10.15694/mep.2021.000129.1

**Published:** 2021-05-15

**Authors:** Alice Alexander, Manisha Singh, Angela Scott, Rebecca Moreira, Timothy Atkinson, Ruth Fissel, Sara Tariq, Sowmya Patil

**Affiliations:** 1 University of Arkansas for Medical Sciences

**Keywords:** Bias, discrimination, racism, students, communication

## Abstract

This article was migrated. The article was marked as recommended.

Physicians in training may experience harassment and discrimination from supervisors, consultants, colleagues, or patients and families. Instances of discrimination towards students may impact students’ self-esteem, self-efficacy, and ultimately performance. In this particular time, many institutions are looking to enhance their curriculum regarding bias. More tools are needed to help students feel empowered to respond professionally when they encounter challenging situations.

This study was designed to assess the impact of a training intervention in addressing biased patient statements. The training was strategically placed prior to clinical interactions. The authors’ intention was to present discriminatory statements by patients as one of the many difficult clinical situations that students are being trained to navigate. The authors developed a clinical rubric for decision-making in flowchart style to mimic the decision trees used in diagnostic or treatment decisions. They then created a workshop to help learners use the flowsheet. The workshop was delivered to third-year medical students as part of “Junior Bootcamp,” a day-long session of events to orient students to the clinical experiences of the M3 year. The workshop was delivered in the summer of 2019.

Respondents indicated that they were more likely to be able to appropriately respond to discriminatory comments after completing the workshop. They also felt that they were more likely to be able to engage in respectful dialogue with a patient and to debrief with a faculty member. Fewer participants felt that they were likely to use the flowchart.

Findings indicate that the workshop was useful to participants. It may be especially useful to educators dealing with the COVID pandemic because it is scalable and easily delivered remotely. Further studies are needed to determine if introducing this topic in the clinical years of medical school leads to improved skill in addressing instances of bias that come from patients and families.

## Introduction

Physicians in training frequently experience harassment and discrimination from supervisors, consultants, colleagues, or patients and families. A significant number of instances of bias directed towards trainees come from patients and their families. In a survey of US residents, Baldwin, Daugherty, and Rowley (1994) noted that 23% of residents had reported racial or ethnic discrimination during their training; half of these instances were ascribed to patients. A more recent meta-analysis conducted by Fnais *et al.* (2014) showed that 59% of medical trainees experienced some sort of harassment or discrimination during training; 22% of these instances were ascribed to patients and families.

Healthdisparities, racism, and microaggressions are addressed in mainstream medical training curricula ( Brooks, Rougas & George, 2016; DallaPiazza *et al.*, 2018), but these curricula do not give students a specific framework for dealing with discriminatory comments from patients. Paul-Emile *et al.* (2016) have laid out a decision tree that addresses the ethical implications of a patient’s discriminatory request and Whitgob, Blankenburg, and Bogetz (2016) outlined strategies for addressing discriminatory comments while trying to maintain a therapeutic relationship. We expand on these strategies by providing a focused clinical decision-making framework designed to assist learners in the moment. Simulation training, including didactic and debriefing components, has been shown to help prepare residents to respond to discriminatory comments in the workplace ( March *et al.*, 2018; Eisenberg and Kieffer, 2019). Given the significant prevalence of instances of discrimination during clinical experiences, we designed an intervention to capture learners at the beginning of their clinical years and give them a structured approach to these difficult situations. With the recent spotlight on implicit bias and structural racism, students are looking to their institutions to cover this material in a robust way.

## Methods

The institutional review board at our institution determined that the project was exempt from IRB review.

Third-year medical students from the University of Arkansas for Medical Sciences who participated in a 2019 training workshop on discrimination and completed a pre- and post-survey were included in the study. The workshop was part of “Junior Bootcamp,” a day-long session of events to orient the third-year medical students at the beginning of their clinical rotations. Students who did not complete both surveys were excluded from the analysis.

The University of Arkansas for Medical Sciences (UAMS) is a tertiary care academic center located in a central, capital city in a predominantly agricultural southern state. We have 174 medical students. The institution is significantly more diverse than the communities within its large catchment area. The majority of students in the College of Medicine are from Arkansas. The medical school uses a traditional curricular model with the first two years being primarily pre-clinical and the third and fourth years being primarily clinical.

Based on events at our institution, a multidisciplinary workgroup composed of five physician faculty members, a social worker, and a medical student started meeting in May 2018 to address instances of discrimination and bias towards students. Our institution has a global policy about bias and discrimination, but we do not have a specific policy about discriminatory requests from patients. We reviewed the available literature on educational approaches and tools to respond effectively to discriminatory statements. Our literature review suggested that these instances occur commonly in teaching settings, but no specific tool existed to prepare students, trainees or faculty to effectively and professionally respond to them in the moment. Students who shared experiences of discriminatory statements by patients described a conflict between a personal ethic of self-respect, which called for a response to inappropriate comments, and a professional norm of self-sacrifice, which seemed to suggest it would be unprofessional to respond.

We created a clinical rubric for decision making in flowchart style to mimic the decision trees used in diagnostic or therapeutic decisions. Our intent was to present discriminatory statements from patients as one of the many difficult clinical situations the students are being trained to effectively navigate. We set out to provide a set of responses and rubric for decision making to enable students to respond to a situation in which they may feel not only professional stress but also personal disempowerment.

**Figure 1.**
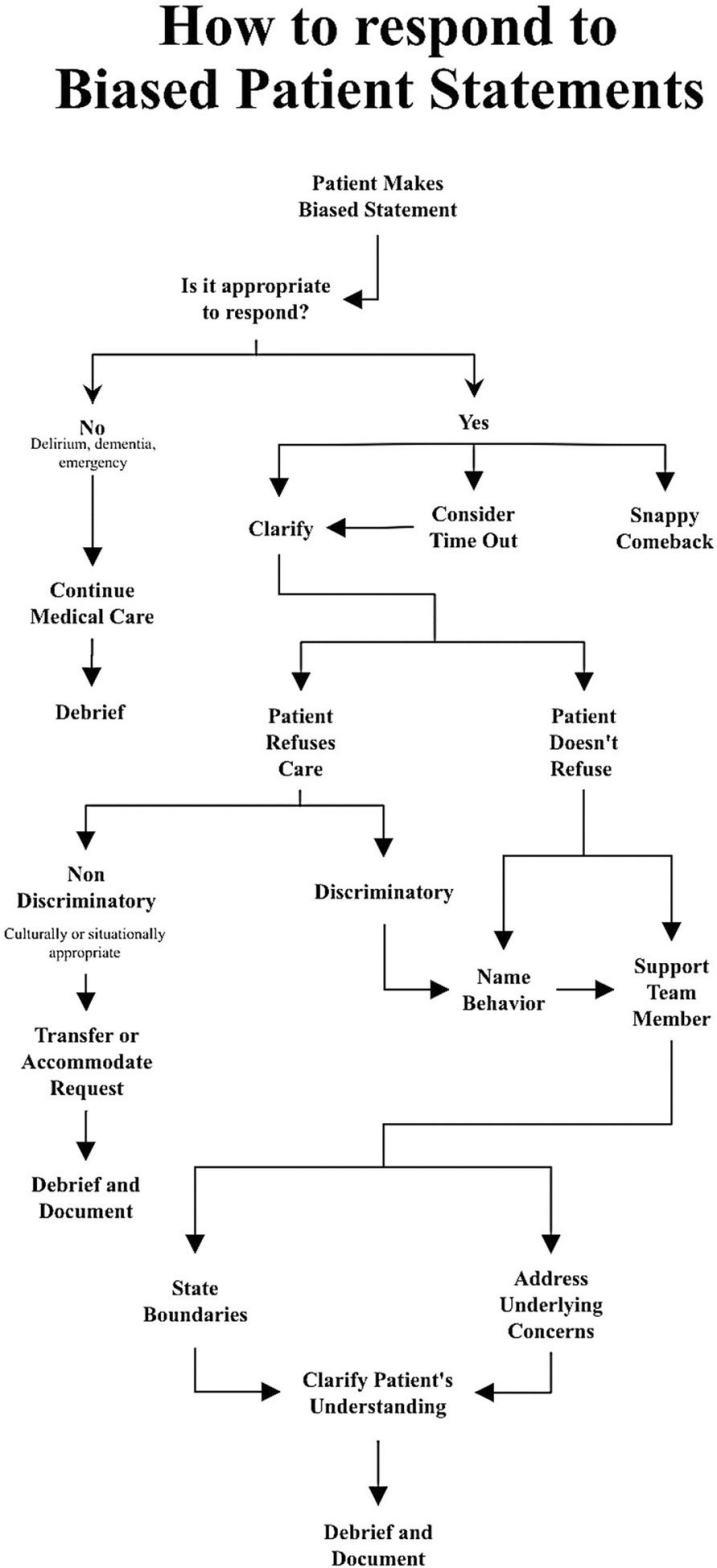
Flowsheet for medical students to address bias

Based on the approaches outlined by Whitgob, we developed a flowsheet ( Figure 1) designed to address a broad spectrum of potentially discriminatory statements. The underpinnings of the approach involved the following: evaluating the clinical acuity of the situation, determining the ethical appropriateness of a response, maintaining the doctor-patient relationship, and supporting the learner.

We then created a workshop to help learners use the flowsheet. Our first workshop audience was a group of educators at an institutional “Teach the Teacher” conference. We included some background information about bias and the impact of bias, and we then walked through each of the steps in the flowsheet. We discussed the spectrum of discriminatory statements and gave specific examples of possible “comebacks” for more minor comments. We explained how to clarify discriminatory intent and discussed situations in which a request to change providers might be ethically appropriate. We included statements of support for team members and gave examples of empathetic statements that could help address a patient’s underlying concerns about his or her care. After the didactic session of the workshop, learners worked in small groups to apply each of the flowsheet steps to a sample case. We then led a debrief session and solicited responses from individuals in the audience.

Our subsequent workshop was delivered to third-year medical students as part of “Junior Bootcamp,” a day-long session of events to orient students to the clinical experiences of the M3 year. To determine the impact of the workshop, we had students fill out an immediate pre- and post-survey regarding their previous experiences with bias and their comfort level addressing bias directed towards them from a patient. The complete survey is available as Supplementary File 1.

## Results

Among the 2017 matriculants, 54% are male and 46% are female. Matriculants are 74% white, 7% African-American, 12% Asian, 3% Hispanic, 2% Native American, and 1% declined to respond. Average age of matriculants is 22.6 years (range 21-35).

Pre-workshop survey participants totaled 144, while 125 respondents completed both the pre- and post-workshop questionnaires. For a response rate, 86% of participants completed both the pre and post-workshop surveys. Items were rated using a five-point Likert scale (1 = not at all likely, 2 = minimally likely, 3 = moderately likely, 4 = likely, 5 = extremely likely). Wilcoxon signed ranked test (a non-parametric paired samples t-test) results revealed no differences in results when missing values were removed from the calculations, therefore the study team removed missing values from the final results.

**Figure 2.**
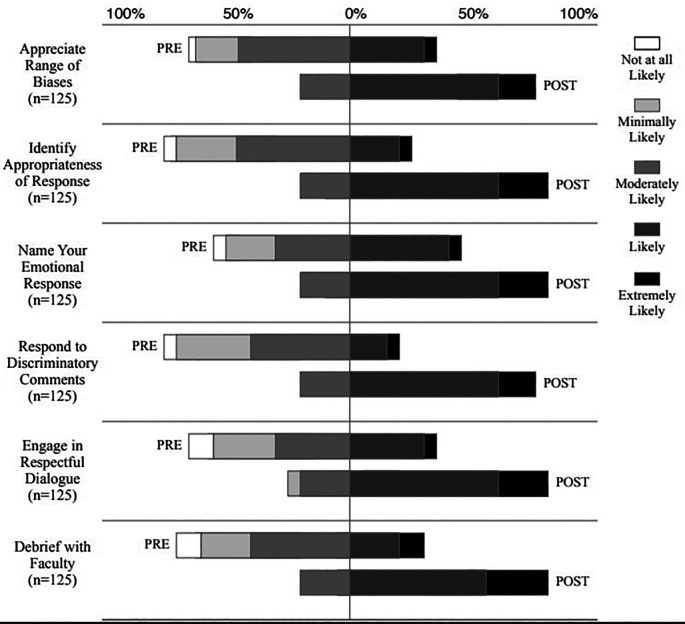
When We Face Bias Workshop, pre- and post-workshop percentage scores

For the final, complete respondent group (N = 125), data were ordinal and not normally distributed, which prompted the use of a non-parametric, paired samples t-test (Wilcoxon) on five survey parameters, pre- and post- workshop intervention. Wilcoxon signed ranks tests illustrated median post-test ranks statistically significantly higher than the median pre-test ranks for all six questions (p <0.001) ( Figure 2), which demonstrated a positive change in participant confidence to handle bias after the workshop.

Further, Kruskal-Wallis analysis indicated no significant difference in pre-workshop confidence scores between those who had experienced bias (29.8%) and those who had not experienced bias (70.2%). In addition, Kruskal-Wallis analysis revealed a statistically significant difference (p <0.001 in pre-workshop confidence scores) between respondents who felt their medical training adequately prepared them to handle bias (54.3%) and respondents who felt medical training had not prepared them (45.7%), on all items except “Name Your Emotional Response”. Post workshop scores between these groups were not significantly different across all questions, indicating a consistent training effect.

The qualitative prompt “What is the nature of the bias?” revealed the common theme of medical learners experiencing the biased assumption by patients that they were a nurse. Because 10 of 17 of the *in vivo* responses dominated this narrative, the team decided *in vivo* codes were enough to reach this conclusion without engaging in further qualitative coding stages.

**Figure 3.**
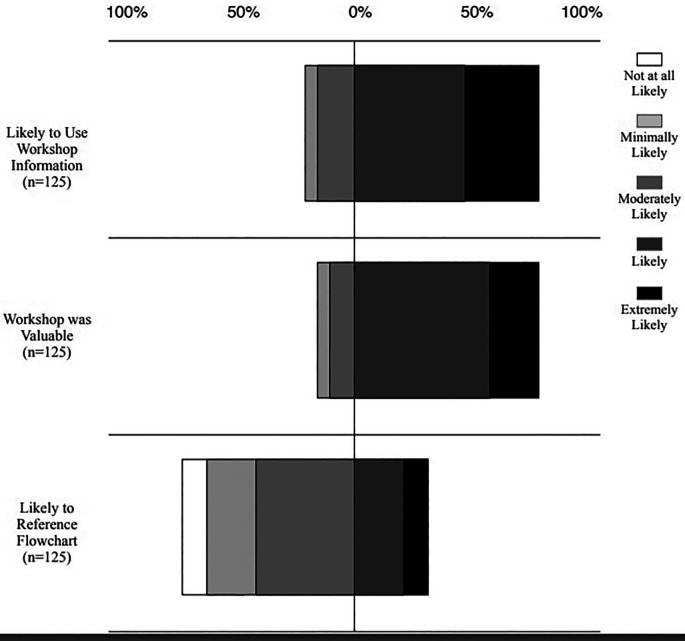
Survey to determine how likely individuals would be to use the information learned in the workshop

Furthermore, 82.4% of post-workshop respondents found the workshop extremely valuable, while 81.6% of post workshop respondents indicated an extremely likely chance they would use the information to handle bias. Overall, respondents were more likely than not to reference the flowchart tool to help them address issues of bias ( Figure 3).

## Discussion

This study provides a powerful, accessible tool for beginning clinical learners to respond in the moment to discriminatory comments from patients. It adds to the existing literature because it demonstrates that it is feasible to introduce this topic in a large-group setting to a medical student audience. In addition, it provides a tangible tool for handling bias in emotionally charged situations. Medical students felt that the workshop was useful and relevant to them.

Our work adds to the international conversation that is currently happening about how to mitigate and address bias. Our approach focuses on challenging bias while working on maintaining the patient-physician relationship. Because learners may incur psychological damage from instances of bias, we wanted to provide both validation of their experiences and tools to deal with potential situations. Previous published approaches for dealing with biased patients have focused on training resident physicians; our intervention focused on introducing this topic prior to clinical encounters.

While there is little published data linking specific instances of discrimination from patients to adverse outcomes for medical students, there is data about how adverse experiences during medical school affect students’ well-being. Witnessing instances of discrimination towards other medical students is associated with increased depressive symptoms, irrespective of the student’s racial or ethnic background ( Hardeman *et al*., 2016). Approximately 30% of the students in our study reported facing bias in their medical training to date, demonstrating the importance of teaching students about bias early in their clinical careers. As clinical exposure increases, so do exposures to discriminatory patients and families: 71% of pediatric residents in one study had witnessed a discriminatory comment from a patient or family ( March *et al*., 2018) and 79% of internal medicine residents in another study (Eisenberg and Kieffer, 2019) had witnessed such a comment directed towards a team member. Discriminatory behavior by a patient towards a resident physician may contribute to feelings of poor self-esteem, self-efficacy, and ultimately even poor performance ( Reynolds *et al.*, 2015).

Orientationto third year provides a critical window to address developing professional identity; our workshop format also provides a “safe space” in a way that simulation exercises may not. Creating psychological safety within a simulation exercise requires careful attention to pre-briefing and debriefing ( Gaba, 2013); for our early clinical learners, we chose to explore this challenging topic in the context of a workshop and group discussion, rather than a setting in which they would have to “perform” a difficult skill.

Strengths of the study include that the workshop materials are easily transferable and scalable to other medical school settings. This content is also applicable to other health professions trainees. The workshop is easy to deliver online, which is useful to educators who are adapting to distance learning. Our data indicate that learners are willing to use the flowchart. To our knowledge, this is the first comprehensive flowchart that can be used by learners when a patient is exhibiting bias. A limitation of our study is that the workshop was given at a single institution. More studies are needed to determine if the training results are generalizable. We also need to ensure that the flowchart is easily accessible, perhaps on a pocket card or as a file on the student affairs webpage. Our review of the literature reveals a paucity of evidence about the detrimental effects of bias and discrimination on medical trainees; this is also an area for future research.

Successful implementation of these tools by students relies on faculty development in this area. We are presenting the workshop to faculty, residents, and students beyond the 3rd year. The training is included in the course catalog of our institutional Division of Diversity, Equity, and Inclusion. In addition, this curriculum has increased awareness about the problem of bias against healthcare workers and has garnered support and interest from key leadership at our institution. Our work has paved the way for a more comprehensive institutional policy about discriminatory requests from patients.

## Take Home Messages

• Students encounter bias early in their medical eduction.
• It is therefore important to introduce bias training early in medical education.
• Providing a tangible communication tool can help medical students navigate challenging patient encounters.
• Students need a repository of “just-in-time” responses to address instances of bias.
• Every institution should have a policy to address discriminatory requests from patients.

## Notes On Contributors

**Alice Alexander** is an associate professor in the Department of Internal Medicine and the Department of Pediatrics, University of Arkansas for Medical Sciences, Little Rock, AR.

**Manisha Singh** is an assistant professor in the Department of Internal Medicine, University of Arkansas for Medical Sciences, Little Rock, AR.

**Angela Scott** is an assistant professor in the Department of Pediatrics, University of Arkansas for Medical Sciences, Little Rock, AR.

**Rebecca Moreira** is a medical student at the University of Arkansas for Medical Sciences, Little Rock, AR.

**Timothy Atkinson** is an assistant professor in the Department of Internal Medicine, University of Arkansas for Medical Sciences, Little Rock, AR.

**Ruth Fissel** is the program manager for behavioral health in the Integrated Medicine Service Line, University of Arkansas for Medical Sciences, Little Rock, AR.

**Sara Tariq** is a professor in the Department of Internal Medicine, University of Arkansas for Medical Sciences, Little Rock, AR.

**Sowmya Patil** is an associate professor in the Department of Pediatrics, University of Arkansas for Medical Sciences, Little Rock, AR.
