# Supplementary material for: Empowering Medical Students to Respond to Discriminatory Comments from Patients: A Just-in-Time Training Method
Source: MedEdPublish (2016). 2021 May 15;10:129. [Version 1] doi: 10.15694/mep.2021.000129.1 (PMC10939538; doi:10.15694/mep.2021.000129.1)
Supplement: Peer review history

# Reviewer report: Reviewer response for version 1

**DOI:** 10.21956/mep.18910.r27025

**Published:** 2021-07-14

**Authors:** Richard Hays

**Affiliations:** 1 James Cook University

This review has been migrated. The reviewer awarded 4 stars out of 5 I found this a useful article to read and to raise in discussions with colleagues. I found the flow sheet helpful in provoking discussion and thinking of examples of behaviours that may appear to be discriminatory. The evaluation results are strong, although a response rate is not mentioned and evaluation a few weeks later may be more meaningful as participants would have time to reflect and even observe the relevance of the flowsheet. I have a question for the authors: should a behaviour in a non-urgent situation be found to be discriminatory and the patient does not refuse treatment, how is the potential for escalation of conflict through naming the behaviour managed? How can this be made educational for the patient? I note the strong support for colleagues, which is vital, but but also see the potential to change discriminatory behaviours and perhaps even opinions. Another point is that I do not see how this workshop fits my understanding of 'just in time' product - a supply chain produce that is supplied when needed. This kind of learning experience should be offered to all health professional students and then re-inforced as required through debriefing after concerning incidents.

Reviewer Expertise:

NA

No decision status is available

# Reviewer report: Reviewer response for version 1

**DOI:** 10.21956/mep.18910.r27024

**Published:** 2021-05-18

**Authors:** Balakrishnan Nair

**Affiliations:** 1 Centre for Medical Professional Development and University of Newcastle

This review has been migrated. The reviewer awarded 4 stars out of 5 Dealing with discriminatory comments is a significant challenge for the learner. This project is a good initiative. We have multi-racial learners and patients. Most medical schools have more female students. Students should anticipate, prepare and deal with discriminatory comments. Training them and preparing them is very timelyThe learners found the workshops very useful and helped them to attain "professional identity" and "safe space". This learning is very vital for them and will help them in the long term. Teaching them to respond " just in time" is very importantIt will be interesting to study the long term effect of this.

Reviewer Expertise:

NA

No decision status is available
